# Stress Distribution in Silicon Subjected to Atomic Scale Grinding with a Curved Tool Path

**DOI:** 10.3390/ma13071710

**Published:** 2020-04-06

**Authors:** Xudong Fang, Qiang Kang, Jianjun Ding, Lin Sun, Ryutaro Maeda, Zhuangde Jiang

**Affiliations:** 1 School of Mechanical Engineering, Xi’an Jiaotong University, Xi’an 710049, China; dongfangshuo30@xjtu.edu.cn (X.F.);; 2 State Key Laboratory for Manufacturing Systems Engineering, International Joint Laboratory for Micro/Nano Manufacturing and Measurement Technology, Xi’an Jiaotong University, Xi’an 710056, China; 3 Shaanxi Key Laboratory of Non-traditional Machining, Xi’an Technological University, Xi’an 710021, China; 4 Institute of Mechanical System Engineering, The National Institute of Advanced Industrial Science and Technology (AIST), Tsukuba 3058564, Japan

**Keywords:** molecular dynamics, stress distribution, curved tool path, atomic grinding

## Abstract

Molecular dynamics (MD) simulations were applied to study the fundamental mechanism of nanoscale grinding with a modeled tool trajectory of straight lines. Nevertheless, these models ignore curvature changes of actual tool paths, which need optimization to facilitate understanding of the underlying science of the machining processes. In this work, a three-dimensional MD model considering the effect of tool paths was employed to investigate distributions of stresses including hydrostatic stress, von Mises stress, normal and shear stresses during atomic grinding. Simulation results showed that average values of the stresses are greatly influenced by the radius of the tool trajectory and the grinding depth. Besides the averaged stresses, plane stress distribution was also analyzed, which was obtained by intercepting stresses on the internal planes of the workpiece. For the case of a grinding depth of 25 Å and an arc radius 40 Å, snapshots of the stresses on the X–Y, X–Z and Y–Z planes showed internal stress concentration. The results show that phase transformation occurred from α- silicon to β- silicon in the region with hydrostatic stress over 8 GPa. Moreover, lateral snapshots of the three-dimensional stress distribution are comprehensively discussed. It can be deduced from MD simulations of stress distribution in monocrystalline silicon with the designed new model that a curved tool trajectory leads to asymmetric distribution and concentration of stress during atomic-scale grinding. The analysis of stress distribution with varying curve geometries and cutting depths can aid fundamental mechanism development in nanomanufacturing and provide theoretical support for ultraprecision grinding.

## 1. Introduction

Stress distribution during the machining of silicon has a big effect on the properties of the final product, which deserves further study. Monocrystalline silicon is a substrate for continuously manufacturing miniaturized parts of microelectromechanical systems (MEMS), integrated circuits and optical components [1,2]. Grinding, as one type of ultraprecision machining methods, is broadly applied to process this material [3]. Related research has been ongoing for decades in fundamental mechanism revelation and engineering technology development [3,4]. One main method is by investigating the relationships among structure, processing and properties. This work is more commonly executed with advanced characterization tools, which makes it possible to measure material properties at the nanoscale, e.g., by scanning probe microscopy (SPM) and atomic force microscopy (AFM). Among the parameters studied for silicon, stress is an important parameter which has a great influence on fatigue damage, surface cracks, phase transformation and structural deformation [4,5], and has been vigorously investigated.

Nevertheless, online investigations of stress distribution in nanoscale or atomic-scale grinding for fundamental research is challenging, and reports thereof are few. The stress, defined as internal force per unit area in a workpiece, is mainly affected by machining forces and temperature variations. It will occur in the region near the machined surface. For instance, in nanomanufacturing, microcompressive stress or tensile stress forms on the workpiece surface induced by thermal dissipation from bonds breaking among atoms [6]. Generally, the internal stress is in an equilibrium state. If the self-balancing state is destroyed by an external force or temperature field, the residual stress—which affects local plastic deformation and surface defects—remaining on the workpiece after machining will be changed [7]. This mainly includes compressive and tensile stress after grinding. Large tensile residual stress compromises the performance of the workpiece, and may increase the probability of fatigue cracking or fracture. In contrast, compressive residual stress is beneficial to enhancing structural integrity and durability [8,9], which is beneficial to the functional performance of the final component. Accordingly, contemporary techniques usually seek to increase compressive residual stress in the field of ultraprecision machining by trial, such as polishing, shot peening, abrading and heat treatment [9,10]. However, the fundamental mechanism of residual stress generation and evolution is still not clear. A growing consensus is that internal stress during machining has serious effects. Accurate measurement of the magnitude and distribution of internal stress is indispensable to adjust engineering processes and select optimal grinding parameters. Due to the limitations of existing hardware, online measurements of internal stress during grinding are unavailable. 

With development of technology and the increasing demand for atomic scale precision, molecular dynamics simulations are used to explore the fundamental mechanism of stress distribution and evolution. Some experimental methods are effectively applied to detect static stress in macrostructures, such as optics, neutron diffraction, ultrasound, X-ray and electromagnetism [11]. Nevertheless, they are not suitable for online measurement because of the limited resolution of current devices [12,13]. Consequently, researchers have tried to use theoretical tools to understand the mechanism at the atomic scale to better control residual stress. For ultraprecision machining at the atomic scale, the removal thickness of the silicon workpiece is only a few atomic layers. The magnitude of the stress depends to a large extent on the processing parameters, surface morphology and the machining direction. Because of the complexity of the grinding process, it is extremely difficult to describe stress distributions with an analytical mathematical model. MD simulation has been shown to be a crucial tool in the study of nanomachining [14]; it can provide an effective microscopic approach to calculate stresses of each atom by time integration of Newton’s second law [15,16]. 

The stresses during grinding, including hydrostatic, von Mises, normal and shear stresses, have been studied via MD simulation, but few researchers have considered the curvature of the tool paths. The interrelation of stress distribution with temperature distribution, volume deformation and phase transformation has been revealed. For instance, Li et al. employed MD simulation to establish a three-dimensional model (3D) of diamond cutting single-crystal silicon [17]. A 3D arrangement of hydrostatic and von Mises stresses on the X–Y plane was observed. Considering the effects of stress and temperature, the relationship among subsurface dislocation, plastic transformation and crystal damage was studied. Dai et al. established a model to analyze the distribution of residual stresses, focusing on *σ_xx_*, *σ_yy_* and *σ_xy_* in the X–Y plane; they investigated subsurface damage and inelastic deformation patterns [18]. The results showed that less compressive normal stresses *σ_xx_* and *σ_yy_* occurred when machining brittle silicon with a structured atomic-scale tool. In another work using MD to study stress distribution of silicon material, the findings showed that hydrostatic pressure damages the lattice structure and causes phase transformation [19]. Additionally, nonuniformity of the lattice structure causes concentration of tensile stress in the modified layer. As for shear stress, a dynamical model in amorphous solids at low temperature was put forward by Falk et al. using MD simulation [20]. For metallic glass, the typical mechanism of reversible elastic deformation under small stress and irreversible plastic deformation under large stress was revealed. Recently, residual stress on the surface of monocrystalline silicon after machining with a straight path was studied using MD simulations [9]. It was predicted that with increased cutting depth, the maximum tensile residual stress would decrease, while the maximum compressive residual stress showed reverse trend. Similar work was done to study stress variations of *σ_xx_*, *σ_yy_* and *σ_xy_* in a deformation zone [21]. The stresses almost simultaneously reached the peak values in this zone. Based on the above literature, MD simulations were proved to be an effective tool for online stress distribution analysis at the atomic scale. Even so, one common issue with the above MD simulation models is that tool trajectory is a straight line [16,17,18,19], i.e., curvature is not considered. 

Stress distribution during machining considering the effect of tool paths needs investigation. Various methods were used to generate tool paths for grinding to obtain high precision parts with the desired geometries [22]. Macroscopically, all the tool paths generated have curvature changes to finish the grinding process, such as helical and elliptical shapes [23,24,25]. Using the most common tool path, i.e., a circular shape, as an example, if the geometry of the model is shrunk to the atomic scale, the scratch track of the diamond grains when machining should not be a simple straight line. However, in the literature [15,26,27], the diamond tool trajectory is not curved. One possible reason for this is that the degree of stress variation during nonlinear grinding is more complicated than linear grinding, even though it is closer to the actual situation. The simulation model of a straight path for grinding monocrystalline silicon is relatively easier to establish. Due to limitations in the available software, it is difficult to complete the modeling of a curved trajectory with the available tools. As a result, stress evolution changes due to curved tool paths cannot be obtained, and the effect on the material’s properties and structure needs further exploration. 

Fundamental research on grinding-induced stress distribution plays an important role in nanomanufacturing and its applications. However, either conducting an actual experiment or testing the workpiece properties at the nanoscale is a quite challenging task. As online measurement approaches are rare for stress during grinding, available experimental studies yield rather scattered static data. Consequently, MD simulation was selected in this work to carry out in-depth studies of stress evolution during grinding with a curved tool path and of the subsequent residual stress distribution. A postprocessing programming method was used to realize the curve path grinding. Additionally, a reasonable visualization method was developed to accurately determine the stress distribution during nanocurve grinding. 

In this work, the authors explore hydrostatic stress, von Mises stress, normal stresses and shear stress in the grinding process of a curve trajectory by MD. The effect of grinding depth on stress distribution is studied. The plane stress distribution of the internal atoms was also obtained by intercepting the internal planes of the workpiece, which made it possible to determine the stress concentration in the workpiece during atomic-scale grinding. Moreover, this online simulation of curve path grinding plays an important role in disclosing the subsurface damage caused by mechanical processing and property variations in the material. The results in this work will enrich fundamental mechanism research of nanomanufacturing.

## 2. Simulation Methodology 

### 2.1. MD Simulation Model

**Figure 1:**
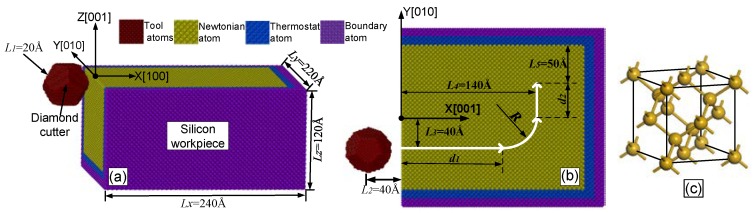
MD simulation setup: (**a**) 3D model; (**b**) movement trajectory design of the diamond tool during grinding; (**c**) schematic diagram of the unit cell of monocrystalline silicon.

A 3D model of orthogonal machining configuration was adopted to simulate the atomic-scale grinding process, as shown in Figure 1a. The simulation parameters applied in this model are summarized in Table 1. The geometric model is composed of a monocrystalline silicon workpiece and a rigid diamond tool. A Cartesian coordinate system was established on the workpiece to accurately describe the tool trajectory, in which the three orientations ([100], [010] and [001]) are the X-, Y- and Z-axes, respectively. The silicon model with dimensions of 240 Å × 220 Å × 120 Å contains 315,414 atoms, or more specifically, three types of atoms: the innermost Newtonian layer, the outermost boundary layer and the intermediate thermostatic layer [28,29,30]. These three layers are represented by yellow, purple and blue, respectively. The Newtonian layer is the simulation zone during nanogrinding. The motion of the atoms in this layer obeys Newton’s second law, as determined by the integral of the Hamilton equation with a time step of 1 fs. The thermostat layer dissipates heat generated in the simulation zone and maintains a stable temperature of 293 K under canonical ensemble (NVT). That is similar to the heat transferred to the coolant, grinding wheel, chips and the atmosphere in macrogrinding [16,29]. The berendsen command was used to adjust the temperature of the atoms in the thermostat layer. The fixed boundary layer was used to reduce influence of the boundary and support atoms in the moving region [13]. Figure 1c shows a schematic diagram of the unit cell of monocrystalline silicon, which has 18 silicon atoms. This cell is a face-centered cubic (FCC) structure like that of the diamond. Such cells formed the macroworkpiece. The 3D geometry of the diamond tool was a regular dodecahedron with a side length of 20 Å; it contained 10,930 atoms. The periodic boundary condition was set as the Z-axis to reduce the influence of the boundary effect [12,15,30]. All simulations of interatomic interactions were calculated by the software—large-scale atomic/molecular massively parallel simulator (LAMMPS). A selection of the interaction potential function between atoms directly determined the accuracy of the atomic stress tensor. In this paper, a Tersoff type potential function was applied to express the atomic interaction within atoms, mainly including Si–Si, Si–C and C–C [15,31,32].

The simulation procedure was divided into two processes, namely, the relaxation process and the atomic-scale grinding process. Initially, the workpiece was maintained at 293K until equilibrium was achieved. The initial atomic velocity was assigned according to the Maxwell-Boltzmann distribution [12]. After a complete relaxation, the grinding process was initiated and the velocity was maintained at 100 m/s [12]. A top view of the simulated structure is shown in Figure 1b, in which the curved white thick solid arrow is the tool trajectory during grinding. It is different from the straight path in the literature [15,26,27]. The initial center coordinate of the tool on X–Y plane was (−40 Å, −40 Å). Firstly, the moving distance of the tool along the direction of [100] was (L2 + d1). The distance of the tool from the X-axis was L3. Then, the tool moved counterclockwise one quarter of the circumference of radius R until its velocity was along the +Y orientation. At this point, the distance between the centroid of the tool and Y-axis was L4. Finally, the tool moved distance d2along the [010] direction. Under such circumstances, the interval from the centroid of the tool to the upper boundary of the Newton layer was L5. The values of L2, L3, L4 and L5 were constant: 40 Å, 30 Å, 140 Å and 50 Å, respectively. Table 2 lists the grinding distances d1 and d2 corresponding to different arc radii R and center coordinates of the arc.

The interaction force *F_ijx_* acting on atom *i* by atom *j* can be obtained by differentiating potential energy in the X-direction as follows [21]:

(1) $$F_{ijx}=-\frac{\partial V_{ij}}{\partial x_{i}}=-\left[\frac{\partial f_{C}}{\partial x_{i}}\left(f_{R}+b_{ij}f_{A}\right)+f_{C}\left(\frac{\partial f_{R}}{\partial x_{i}}+b_{ij}\frac{\partial f_{A}}{\partial x_{i}}+\frac{\partial b_{ij}}{\partial x_{i}}f_{A}\right)\right],$$

where *x_i_* is the X-axis coordinate of atom *i, V_ij_* is the potential energy between the atoms and *b_ij_* indicates the bond order term in Equation (1). *f_A_* and *f_R_* represent attractive and repulsive pair potential, respectively. *f_C_* is a smooth cut-off function to limit the potential range. A similar method can be applied to calculate *F_ijy_* and *F_ijz_*. The interaction force *F_i_* of atom *i* in the workpiece subjected to other atoms can be expressed as [33]

(2) $$F_{i}=m_{i}a_{i}=m_{i}\frac{d^{2}r_{i}\left(t\right)}{dt^{2}},$$

In Equation (2), *r_i_* represents the distance between two atoms in the domain, and *a_i_* and *m_i_* are acceleration and mass of atom *i*. Components of the interatomic force *F_i_* in the X, Y and Z directions calculated in LAMMPS are *f_ix_*, *f_iy_* and *f_iz_*, respectively.

**Figure 2:**
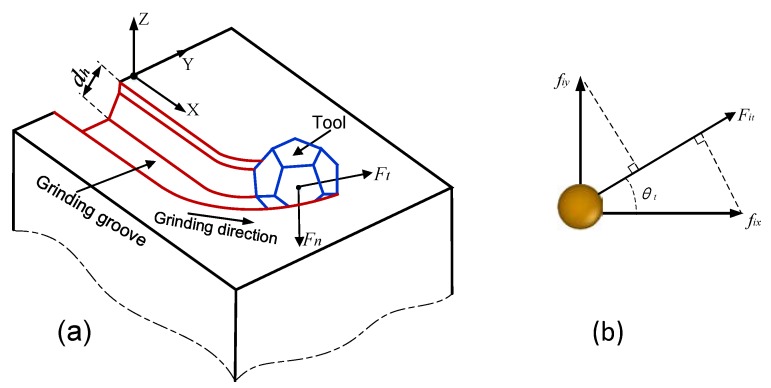
Schematic diagram of the curve grinding process (**a**); the sketch of the tangential grinding force of a single atom (**b**).

The grinding force can be obtained by summing the forces acting on each atom. Figure 2a is a schematic of the curve segment during grinding. The direction of the tangential force *F_t_* is parallel to that of the tool velocity. The front position in the tangential direction of the tool velocity is the shear deformation zone. The tangential grinding force of a single atom in the curve segment on the X–Y plane is displayed in Figure 2b. The normal grinding force *F_n_* and the tangential grinding force *F_t_* can be expressed as

(3) $$F_{n}=\sum_{i}^{N}f_{iz},$$

(4) $$F_{t}=\overset{N}{\underset{i}{\sum }}F_{it}=\overset{N}{\underset{i}{\sum }}\left[f_{ix}cos\left(\theta_{t}\right)+f_{iy}sin\left(\theta_{t}\right)\right],$$

In Equations (3) and (4), *θ_t_* is the angle between tool velocity and X-axis, and *F_it_* is the tangential force of a single atom. 

### 2.2. Simulation Theory of Stress

Generation of internal stress is caused by microstructure uneven volume changes inside the material induced by temperature and external force. Temperature *T* in the local area can be evaluated using the kinetic energy of a single atom, *i*, as follows [34,35].

(5) $$\frac{3}{2}NK_{B}T=\frac{1}{2}\sum_{i=1}^{N}m_{i}\|v_{i}{\|}^{2},$$

In Equation (5), *m_i_* and *v_i_* represent mass and the resultant velocity of atom *i*. *N* is the number of atoms in this local region and *K_B_* is the Boltzmann constant. The stress tensor of each atom has six components in the following order: *S_xx_*, *S_yy_*, *S_zz_*, *S_xy_*, *S_xz_* and *S_yz_*. These components in the surface layer contribute to the formation of the residual stress [9]. The stress tensor of other atoms acting on atom *i* in the workpiece can be calculated using the following formula, where the values of *a* and *b* may be two of *x*, *y and z* [36,37].

(6) $$\begin{array}{cc} S_{ab}= & -[mv_{a}v_{b}+\frac{1}{2}\sum_{n=1}^{N_{p}}\left(r1_{a}F1_{b}+r2_{a}F2_{b}\right)+\frac{1}{2}\sum_{n=1}^{N_{b}}\left(r1_{a}F1_{b}+r2_{a}F2_{b}\right)\\ & \;+\frac{1}{3}\sum_{n=1}^{N_{a}}\left(r1_{a}F1_{b}+r2_{a}F2_{b}+r3_{a}F3_{b}\right)\\ & \;+\frac{1}{4}\sum_{n=1}^{N_{d}}\left(r1_{a}F1_{b}+r2_{a}F2_{b}+r3_{a}F3_{b}+r4_{a}F4_{b}\right)\\ & \;+\frac{1}{4}\sum_{n=1}^{N_{i}}\left(r1_{a}F1_{b}+r2_{a}F2_{b}+r3_{a}F3_{b}+r4_{a}F4_{b}\right)\\ & \;+\mathrm{Kspace}(ri_{a},Fi_{b})+\sum_{n=1}^{N_{f}}ri_{a}Fi_{b}] \end{array},$$

In Equation (6), the first term represents the contribution of atomic kinetic energy. The second term stands for the pairwise energy contribution in which *r1* and *r2* are the positions of the two atoms under the pairwise interaction. *N_p_* is the number of neighbors around atom *i.* The forces on the two atoms are *F1* and *F2*, caused by pairwise interaction. The third term is a similar form of the bond contribution of the *N_b_* bond, to which atom *i* belongs. Similarly, the meaning of *N_a_*, *N_d_*, *N_i_* and *N_f_* are angle, dihedral, improper interactions and internal constraint forces of atom *i*, respectively. The penultimate term for the Kspace contribution is called long-range Coulombic interactions.

For atomic-scale grinding, the workpiece was composed of many discrete silicon atoms. Because of the irregular movement of atoms, and the nonconvergence and divergence of the results calculated by the software, the stress tensor of a single atom can be extraordinarily large or small. Additionally, the stress of a single atom cannot effectively reflect stress distribution in the workpiece. Therefore, in the MD system, the virial stress components of atom *i* can be calculated with the following expression [9,36,38,39].

(7) $$\sigma_{ab}=\frac{1}{\Omega }\sum_{i}^{N}S_{iab},$$

In Equation (7), *σ_ab_* is the average virial stress and $\overset{N}{\underset{i}{\sum }}S_{iab}$ is the stress sum contribution of the *N* atoms in a sphere centered on the atom *i*. The sphere has a radius of the cutoff distance *r* and volume *Ω*. The values of *r* and *Ω* are 2 nm and 33.49 nm^3^, respectively.

In this work, normal stresses (*σ_xx_*, *σ_yy_* and σ_zz_) and shear stress *σ_xy_* are the main study objectives. Additionally, hydrostatic stress *σ_hyd_* and von Mises stress *σ_von_* were investigated as they are closely related to atomic dislocation nucleation, phase transformation and volume deformation. As previously reported, hydrostatic stress is related to volume change which can cause phase transformation of brittle materials from classical thermodynamics [17,18]. It is also an important reason for the destruction of ideal chemical bond interactions that can induce phase transformation from *α*-silicon to *β*-silicon during atomic-scale grinding [9]. The phase transformation under high hydrostatic pressure around the diamond tool caused the silicon workpiece to transform into ductile mode [18]. During atomic-scale grinding, silicon is affected by deviatoric stresses that can cause shear-induced metallization; this refers to the stress that deviates from hydrostatic stress and causes deformation [37,40,41]. The hydrostatic stress *σ_hyd_* can be calculated with the following equation:

(8) $$\sigma_{hyd}=\frac{1}{3}\left[\sigma_{xx}+\sigma_{yy}+\sigma_{zz}\right],$$

Von Mises stress *σ_von_* quantifies shear deformation that controls shape change, which is normally achieved by activating the defect transmission mechanism [42]. *σ_von_* is also called equivalent stress, which can quantitatively evaluate the degree of inelastic deformation in the shear zone [27,43]. The expression of *σ_von_* can be defined as follows:

(9) $$\sigma_{von}=\sqrt{3\left(\sigma_{xy}^{2}+\sigma_{yz}^{2}+\sigma_{xz}^{2}\right)+\frac{1}{2}\left[{\left(\sigma_{xx}-\sigma_{yy}\right)}^{2}+{\left(\sigma_{xx}-\sigma_{zz}\right)}^{2}+{\left(\sigma_{zz}-\sigma_{yy}\right)}^{2}\right]}\;,$$

As observed in Equations (8) & (9), hydrostatic stress and von Mises stress can be calculated with the normal and shear stresses.

## 3. Results and Discussion

### 3.1. Effect of Arc Radius

**Figure 3:**
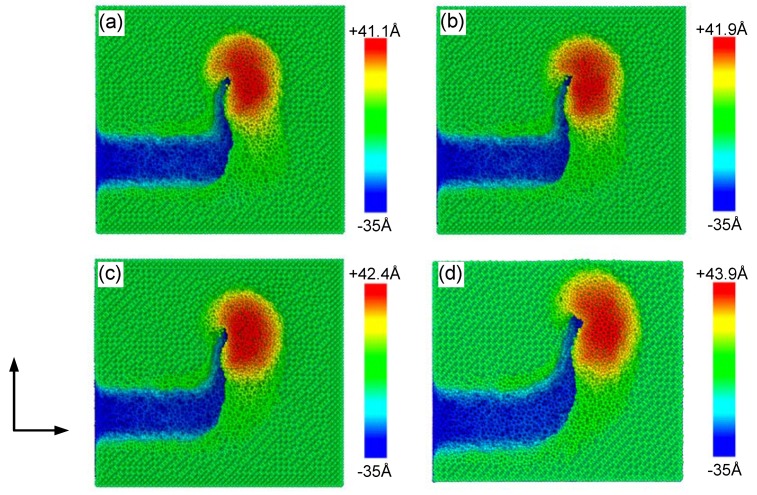
Surface morphology after grinding with different arc radii: (**a**) 0 Å;(**b**) 20 Å; (**c**) 40 Å; and (**d**) 60 Å. The color of the atoms represents their coordinates on the Z-axis.

Atomic scale grinding with a curve tool path was analyzed with a group of radii; the effect of the arc radius on the surface morphology was thus obtained. The grinding depth was fixed at 25 Å and four cases with different arc radii (0 Å, 20 Å, 40 Å and 60 Å) were selected for the tool trajectory. The coordinate of the tool on the X–Y plane was (140 Å, 40 Å). Figure 3 shows snapshots of the arc groove profile and surface morphology of the upper surface after grinding. It can be observed that a region with a high deformation rate was generated around the diamond tool. To intuitively display the outline of the groove and the atoms piled on the upper surface, the subscript of the color scale was set to –35 Å. In the curve segment, the stacked atoms around the tool may fall into the grinding groove due to deviations of the tool from the original trajectory. Little difference exists in the peak value of the atoms piled after comparing the surface morphologies of the four cases. Nonetheless, the piled height increased slightly with the arc radius.

**Figure 4:**
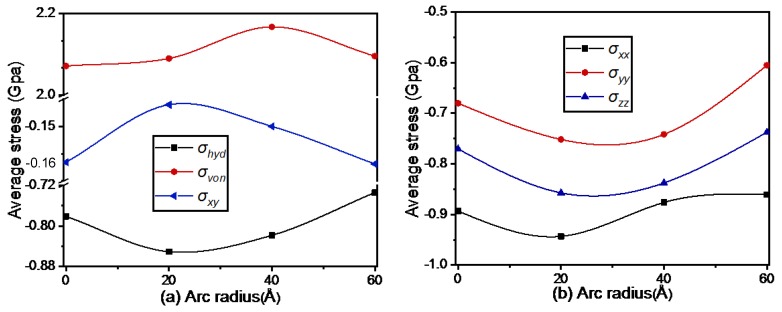
Variation of stresses with arc radius of the tool trajectory: (**a**) hydrostatic stress, von Mises stress, shear stress; (**b**) normal stresses.

The effect of arc radii on stress distribution was also investigated using the aforementioned radius values. One important parameter used to evaluate stress distribution is average stress, which is defined as the averaged stress value per atom. The variation of average stress with arc radii is shown in Figure 4. Quantitative evaluations of the average stresses can make it easier to judge the dominant stress type at a certain stage. According to Equation (9), it was confirmed that the value of the von Mises stress was positive, as shown in Figure 4a, while that of the other stresses depended on whether the unit was under compression or tension. With increase of arc radius, the shear stress declined first and then rose, while the other stresses showed the opposite trend. The valley of the shear stress was at the radius of 20 Å. Similarly, the peak values of normal stresses and hydrostatic stress were obtained with radius 20 Å, while those of the von Mises stress were obtained with an arc radius of 40 Å. This indicated that increasing the arc radius to a certain extent caused the system to generate more heat, which, in turn, generated larger stress. When the arc radius increased further, the grinding distance became smaller at the same end position. The general principle is that the longer the grinding distance, the more heat is generated and the greater the stress. Accordingly, the phenomenon in Figure 4 can be explained. Based on the above analysis, it can be determined that the radius of the tool trajectory has a serious impact on the average stresses.

### 3.2. Effect of Grinding Depth

**Figure 5:**
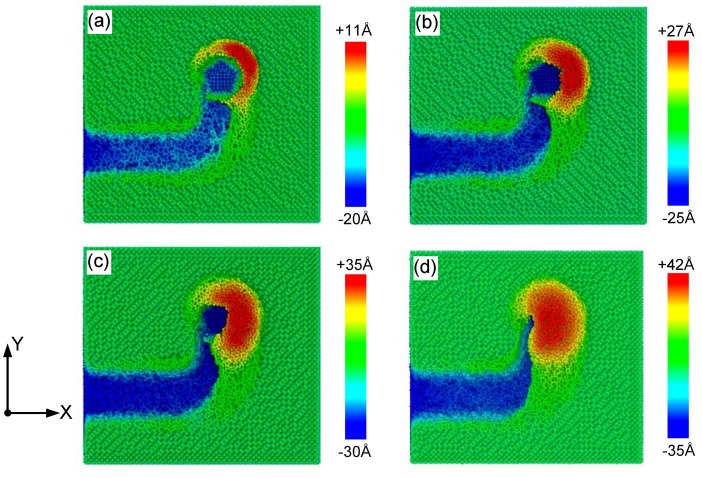
Surface morphologies under the designed grinding depth of: (**a**) 10 Å; (**b**) 15 Å; (**c**) 20 Å; and (**d**) 25 Å after atomic-scale grinding. The colors of atoms were determined by their coordinates on the *Z*-axis.

Another important parameter during grinding is grinding depth. Its impact on surface morphology and stress in a curve tool path needs investigation. The grinding depth was changed from 10 Å, 15 Å, 20 Å to 25 Å to analyze its influence while the arc radius was fixed at 40 Å. Figure 5 shows snapshots of the surface morphology after grinding with different grinding depths *d_h_.* The centroid coordinate of the tool on X–Y plane was (140 Å, 40 Å). The color of the atoms is expressed by coordinates along the Z-axis. The subscript of the color scale was set as the grinding depth plus 10 Å. It can be observed that the grinding depth seriously affects the volume of material pileups on the sides of the arc groove, and that the maximum height of chips increases with the grinding depth. This means that increasing grinding depth can improve the grinding efficiency of brittle silicon.

**Figure 6:**
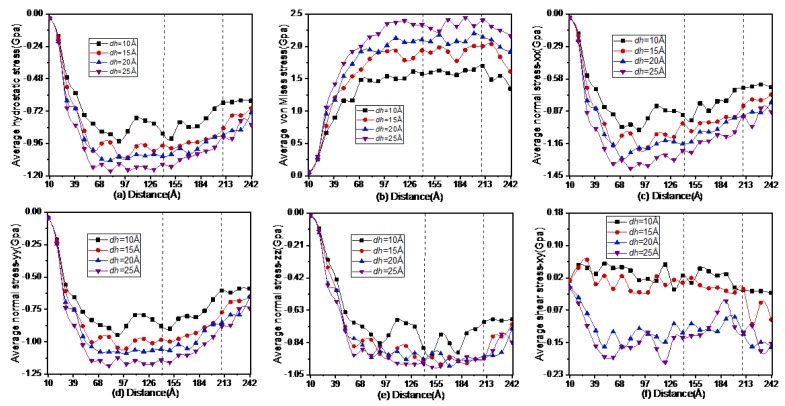
Evolution diagrams of the average stresses including *σ_hyd_* (**a**), *σ_von_* (**b**), *σ_xx_* (**c**), *σ_yy_* (**d**), *σ_zz_* (**e**) and *σ_xy_* (**f**) with the grinding distance under the specified grinding depths.

The effect of grinding depth on stress evolution was studied using the average stresses of the above six types. The results are shown in Figure 6. The term grinding distance includes *L_2_*, *d_1_*, the arc length of radius *R* and *d_2_,* as shown in Figure 1b. The evolution diagrams of the average stresses at the arc segment are shown within the two dashed lines. The right edge of the diamond tool began to grind the workpiece from a grinding distance 13.5 Å. The average values of *σ_hyd_*, *σ_von_*, *σ_xx_*, *σ_yy_* and *σ_zz_* increased rapidly with the grinding distance when it was less than 70 Å and then tended to fluctuate before the first dashed line. The average values of *σ_hyd_*, *σ_xx_* and *σ_yy_* decreased with fluctuations as the grinding distance increased at the arc segment, while *σ_von_* and *σ_zz_* were still in a fluctuating state. The reason for the above phenomenon is that the balance of the entire system was broken as the grinding distance increased after the tool came into contact with the silicon workpiece. The stress distribution in the whole system changed significantly. With competition from energy released from the isothermal layer and atomic bond rupture, the whole system gradually reached a new equilibrium state. When the direction of the tool changes abruptly within the arc, the entire balance may be damaged, and new fluctuations will occur. When the grinding distance exceeded the second dashed line, the average values of *σ_hyd_*, *σ_von_*, *σ_xx_*, *σ_yy_* and *σ_zz_* gradually decreased with oscillations. The average shear stress *σ_xy_* was different, which was obviously negative in some regions under grinding depths of 10 Å and 15 Å, as shown in Figure 6f. It gradually became positive, which indicates compressive stress as the grinding depth increased from 10 Å to 25 Å. Except for an initial increase, the shear stress fluctuated. The reasons for the severe vibration of all the stresses were the uncertainty of the deformation force, thermal energy and dislocation motion [30], while the first two factors were caused by lattice vibrations during atomic-scale grinding.

### 3.3. Stress Distribution

**Figure 7:**
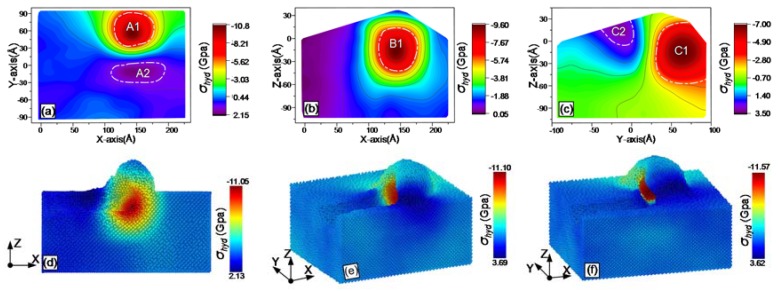
Snapshots of the hydrostatic stress *σ_hyd_* after the atomic-scale curve grinding: (**a**) on X–Y plane; (**b**) on X–Z plane; (**c**) on Y–Z planes; (**d**) at the front point of the arc, (**e**) at the rear of the arc; and (**f**) at the end of the tool.

**Figure 8:**
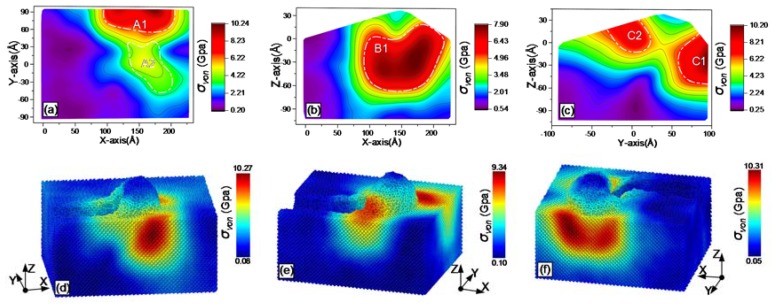
Snapshots of von Mises stress σ_von_ after the atomic-scale curve grinding: (**a**) on X–Y plane; (**b**) on X–Z plane; (**c**) on Y–Z planes; (**d**) at the front point of the arc, (**e**) at the rear of the arc; and (**f**) at the end of the tool.

**Figure 9:**
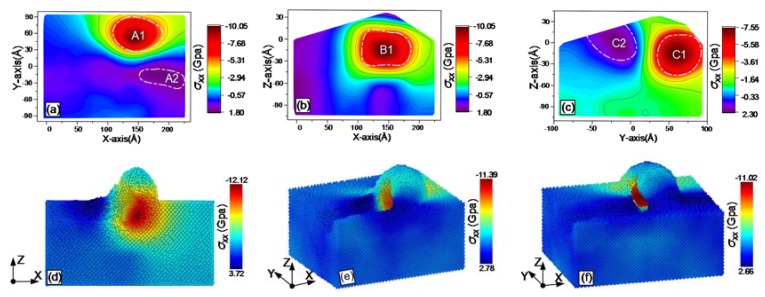
Snapshots of the normal stress σ_xx_ after the atomic-scale curve grinding: (**a**) on X–Y plane; (**b**) on X–Z plane; (**c**) on Y–Z planes; (**d**) at the front point of the arc, (**e**) at the rear of the arc; and (**f**) at the end of the tool.

**Figure 10:**
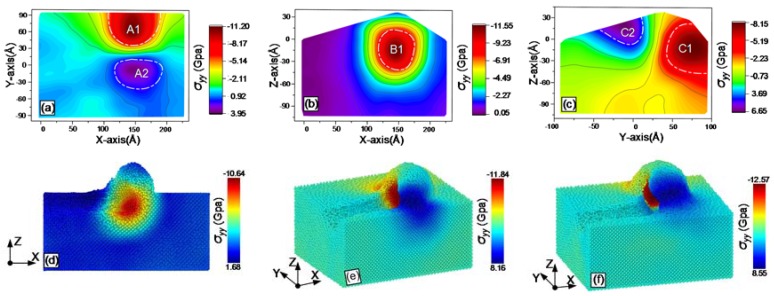
Snapshots of the normal stress σ_yy_ after the atomic-scale curve grinding: (**a**) on X–Y plane; (**b**) on X–Z plane; (**c**) on Y–Z planes; (**d**) at the front point of the arc, (**e**) at the rear of the arc; and (**f**) at the end of the tool.

**Figure 11:**
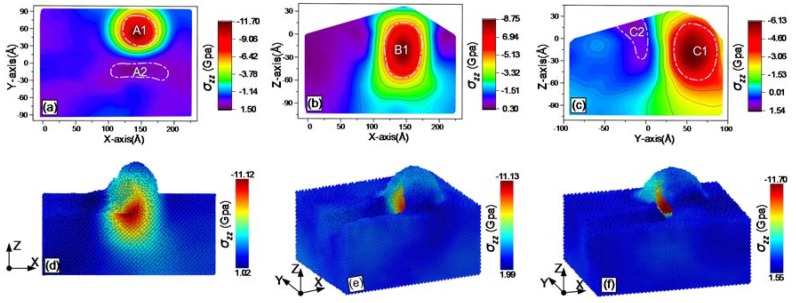
Snapshots of the normal stress σ_zz_ after the atomic-scale curve grinding: (**a**) on X–Y plane; (**b**) on X–Z plane; (**c**) on Y–Z planes; (**d**) at the front point of the arc, (**e**) at the rear of the arc; and (**f**) at the end of the tool.

**Figure 12:**
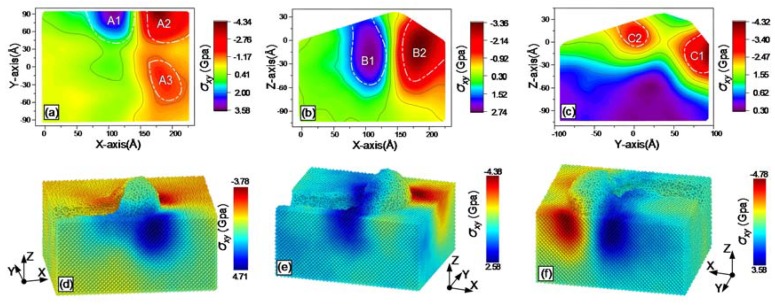
Snapshots of the shear stress σ_xy_ after the atomic-scale curve grinding: (**a**) on X–Y plane; (**b**) on X–Z plane; (**c**) on Y–Z planes; (**d**) at the front point of the arc, (**e**) at the rear of the arc; and (**f**) at the end of the tool.

During grinding, the online stress distribution within the workpiece can be obtained with MD simulations. The results are shown from Figure 7, Figure 8, Figure 9, Figure 10, Figure 11 and Figure 12, including hydrostatic stress *σ_hyd_*, von Mises stress *σ_von_*, normal stresses *σ_xx_*, *σ_yy_* and *σ_zz_* and shear stress *σ_xy_*. Stress values determine the color of the atoms. The center coordinate of the diamond tool was (140 Å, 40 Å) on the X–Y plane. The grinding depth was 25 Å and the arc radius of the tool path was 40 Å. In Figure 7, Figure 8, Figure 9, Figure 10, Figure 11 and Figure 12, Subfigures (a), (b) and (c) show snapshots of the X–Y, X–Z and Y–Z planes, respectively. The stress value of each atom inside the workpiece can be calculated, but it is difficult to clearly see the internal stress distribution. This makes it extremely inconvenient to study the atomic layer structure damage, deformation, fracture and phase transformation in the high stress region inside the workpiece. Thus, through intercepting several typical planes of the workpiece, the stress values of the internal atoms could be displayed using an image post-processing software, i.e., Origin. Subfigure (a) shows the stress distribution of the subsurface layer depth Z = −25 Å on the X–Y plane. Subfigure (b) exhibits stress distribution on the X–Z plane when the Y-coordinate is 68.15 Å. Subfigure (c) is stress distribution at X-coordinate 166.18 Å on the Y–Z plane. The triangular protrusions in the upper end of Subfigures (b) and (c) were due to influence of the atoms piled on the surface. Subfigures (d)-(f) show snapshots of the stress evolution. The three positions represent the front point of the arc, the rear of the arc and the end of the tool, respectively. The center coordinates of the corresponding tools on X–Y plane were (100 Å, −40 Å), (140 Å, 0 Å) and (140 Å, 40 Å). Subfigures (d) in Figure 7, Figure 9, Figure 10 and Figure 11, are cross-sectional views of stress at Y = −40 Å on X–Z plane, while Subfigure (d) in Figure 8; Figure 12, and Subfigures (e) and (f) in Figure 7, Figure 8, Figure 9, Figure 10, Figure 11 and Figure 12 are lateral views of 3D stress distribution. It can be deduced from the stress distribution that the curve trajectory led to asymmetric distribution and a concentration of stress during the atomic-scale grinding, which is different from that of straight-line tool paths. Referring to those reports, the stresses in this work are in the same range, which infers that the simulation method is reliable [19,27,37].

#### 3.3.1. Hydrostatic Stress

Hydrostatic stress distribution after grinding, which affects residual stress formation and material structure change, was studied; the results are shown in Figure 7. High compressive hydrostatic stress of the subsurface layer concentrated in region A1 near the diamond tool, whereas the high tensile hydrostatic stress concentrated in the back region A2 in Figure 7a. In Figure 7b, the high compressive stress region B1 is basically symmetrical to Z = −25 Å; the closer to the convex angle of the diamond tool, the higher the hydrostatic compressive stress. Figure 7c shows that tensile hydrostatic stress was dominant in the region Y < 25 Å, and that the concentrated region was C2. in contrast, the compressive hydrostatic stress occupied a relatively large proportion in the region Y > 25 Å. The stress concentration region was C1. As observed from stress evolution in Figure 7d–f, the range of hydrostatic stress was 3.69 GPa to −11.57 GPa. The high hydrostatic stress zone is concentrated around the diamond tool. During atomic-scale grinding, a phase transformation of atoms occurred, mainly in this high hydrostatic stress region that was squeezed by the diamond tool [44]. When hydrostatic stress exceeded 8 GPa, a phase transformation occurred, i.e., from α- silicon to β- silicon [45]. Meanwhile, the distance between the interactive atoms changed from 2.35 Å to 2.43 and 2.58 Å, accompanied by variation in the number of atoms having coordinate values 5 and 6 in the workpiece [44,46]. Accordingly, the evolution of hydrostatic stress could be a reference for the identification of phase transformation.

#### 3.3.2. Von Mises Stress

The distribution of von Mises stress *σ_von_* after atomic-scale curve grinding is exhibited in Figure 8. A high stress region A1 was generated near the edge of the workpiece. Two peaks are present in A1 in Figure 8a. In the rear of the tool, there was a region A2 with a stress value of approximately 4.22 GPa. It was confirmed that the distribution of von Mises stress in the subsurface layer was different from that of other stresses at the bottom of the diamond tool. In front of the tool, the distribution of von Mises stress on X–Z plane is shown in Figure 8b. The maximum value of the high stress region B1 was 7.9 GPa. On the right side of tool, the distribution of von Mises stress on Y–Z plane is displayed in Figure 8c, with two high stress regions, i.e., C1 and C2. The peak value of the region C1 in front of the tool is evidently higher than that of region C2. As shown in Figure 8b,c, the high stress region was concentrated in front of the tool velocity direction. Additionally, it can be determined from the three-dimensional evolution diagrams in Figure 8d–f that the stress range of all atoms in the workpiece was 0.05 GPa ~10.31 GPa. The peak region of the von Mises stress was not distributed around the tool, but on both sides of the front region of the tool. The value of von Mises stress was closely related to plastic deformation in the shear zone, which needs further investigation for quantitative characterization.

#### 3.3.3. Normal Stresses

The distribution and evolution of normal stresses during grinding with a curve path were obtained using MD simulation; the results are displayed in Figure 9, Figure 10 and Figure 11. In Figure 9a, Figure 10a and Figure 11a, a tensile stress region A2 was present below the region A1 with a concentration of high compressive stress. The compressive stress was due to the fixed side wall atoms. The regions A2 are relatively decentralized in Figure 9a and Figure 11a, while the corresponding region in Figure 10a shows stress concentration. Similarly, high compressive stress regions B1 are shown in Subfigure (b) of Figure 9, Figure 10 and Figure 11. Comparing Subfigure (c) of Figure 9, Figure 10 and Figure 11, the peak of the compressive stress region C1 in Figure 10 is closer to the edge of the workpiece. This was caused by extrusion force generated between the tool in the direction of +Y axis and the atoms in front of the tool. A tensile stress region C2 existed in the chip stacked zone behind the tool. The calculated normal stresses were useful to evaluate phase transformation and microstructure change. When the normal stress produced by the extrusion of the grinding tool was over the critical value of monocrystalline silicon, dislocation nucleation occurred in the lattices. Additionally, brittle-ductile transition was induced by normal stresses in the high compression region during ductile grinding [28].

The evolution of normal stresses during grinding, as observed in Subfigures (d)–(f) of Figure 9, Figure 10 and Figure 11, was useful to check the origin of subsurface damage and structure change. Compressive stress regions were mainly concentrated around the tool, whereas the tensile stress regions were concentrated in the rear of the tool. The peaks of the normal compressive stress and the normal tensile stress for *σ_xx_* gradually reduced during grinding, while the compressive stress for *σ_yy_* slowly increased and the tensile stress changed rapidly from –1.69 GPa to –8.55 GPa. The difference is because the direction of the resultant force by extrusion between the tool and the workpiece changed from the *X*- to the *Y*-axis at the arc segment. For *σ_zz_*, the peak values of compressive stress and tensile stress varies slightly during grinding, as the machining was mainly completed by shear force instead of the normal force of the *Z*-axis to break bonds between atoms. The evolution of normal stresses was consistent with the elastic recovery at the rear of the tool in macroscope, which must be considered when analyzing friction [29].

#### 3.3.4. Shear Stress

The distribution of the shear stress *σ_xy_* is displayed in Figure 12 after the curve grinding. Crack propagation was dominant when shear stress was very low in the chip formation region and dislocation emission could not be maintained. The premise for the occurrence of dislocation emission is that shear stress is beyond the flow stress of the material [21]. A high shear stress region is more likely to generate dislocation emission [18]. In Figure 12a, a positive shear stress region A1 and a negative shear stress region A2 are generated adjacent to the tool. A further peak region of the negative shear stress A3 was also produced behind the tool motion trail. As observed from the distribution of the shear stress on X–Z plane in Figure 12b, the tensile stress region B1 and the negative stress region B2 were near the left and right sides of the tool, respectively. There was a clear demarcation line of low stress region between the region B1 and the region B2. By analyzing the stress distribution on the right side of the diamond tool in Figure 12c, two negative shear stress peak regions C1 and C2 were generated on the front and rear sides of the tool.

The evolution snapshots of the shear stress *σ_xy_* are shown in Figure 12d–f. The left and right sides of the tool were peak regions of the shear stress. Unlike other types of stresses, the maximum value of the negative shear stress and the positive shear stress differed little in magnitude. With changing of arc radius, grinding depth and grinding speed, the peak of shear stresses will vary, which determines crack propagation and dislocation emission inside the workpiece.

Stress distribution analysis during atomic scale grinding was executed using MD simulation. Ultraprecision machining and nanomanufacturing, as the frontier of modern manufacturing technology, can achieve nanoscale or even atomic level precision. The physical phenomena during the machining, such as the scale effect and minimal tool wear, cannot be explained with traditional theoretical models based on continuum mechanics. Molecular dynamics is useful to visually simulate such machining processes as a bridge to connect the micro- and macro- worlds. In this work, stress distribution during atomic scale grinding was simulated using this tool to analyze possible subsurface damage and structure changed due to stress concentration, which cannot be online measured by existing hardware. Compared to previous reports using MD to simulate machining, this work focused on stress analysis and adopted a curve tool path that is closer to practical engineering applications. The effects of curve geometry and grinding depth, i.e., parameters to adjust during realistic machining, were inspected. Accordingly, the location with potential subsurface damage, phase transformation and crack formation could be identified from stress concentrations. For instance, in this work, the region with hydrostatic stress over 8GPa where α- silicon transforms to β- silicon could be found. Similarly, cracks and damage could be predicted from the peak values of the concentrated regions. Even though the simulation could not be directly mapped to practical machining due to scale differences, the results could provide guidance to adjust processing parameters and predict possible damage in the final workpiece to a certain level. The direct-mapping between MD simulation and practical manufacturing needs more time and development of the relevant processing and characterization tools. The main purpose of this work was to study the fundamental mechanism of nanoscale grinding. With the results in this work, it was shown that real-time behavior during deformation including nonuniformity of internal stress can be obtained. It is likely that phase transformation during grinding can be predicted. This cannot be achieved with available experimental devices. Analysis of stress distribution during grinding provides a useful theoretical basis for stress-induced strengthening and crack from the root. 

## 4. Conclusions

In this work, a 3D model considering the effects of tool paths was employed to investigate the evolution and distribution of stresses at the atomic scale by molecular dynamics simulation. Curved geometry was included for the first time, compared to the straight lines used in previous studies, as it is closer to actual tool paths created by numerical control programs during machining. Stress distribution plays an important role in evaluation of subsurface damage, phase transformation and microstructure change during machining. However, due to the lack of online measuring approaches for stress distribution, the results in this work will promote fundamental mechanism development in nanomanufacturing and provide a theoretical support for ultraprecision grinding. The stresses studied included hydrostatic *σ_hyd_*, von Mises *σ_von_*, normal (*σ_xx_*, *σ_yy_* and *σ_zz_*) and shear stresses *σ_xy_*. According to the simulation results, the following conclusions can be drawn:

(1) The effect of arc radii on the surface morphology and stress distribution was investigated. By comparing the surface morphologies of the cases with arc radii (0 Å, 20 Å, 40 Å and 60 Å), it was shown that arc radius plays an important role in determining cross-sectional profiles with the volume of material pileups on sides of the groove. A stress comparison indicated that the shear stress *σ_xy_* declines first and then rises, while the other stresses show the opposite trend.
(2) The effect of grinding depth during curve grinding is studied. For cases with grinding depths of 10 Å, 15 Å, 20 Å and 25 Å, it was shown that a larger grinding depth can improve the grinding efficiency of brittle material silicon at the atomic-scale. The average values of *σ_hyd_*, *σ_xx_* and *σ_yy_* decreased with fluctuations as the grinding distance increased at the arc segment, while the average values of *σ_von_* and *σ_zz_* were in a fluctuating state. The average value of *σ_xy_* was in a state of fluctuation after an initial increase.
(3) Snapshots of the stresses on the X–Y, X–Z and Y–Z planes were analyzed. Additionally, lateral snapshots of the three-dimensional stress evolution during grinding were discussed. It can be deduced from the stress distribution that the curve trajectory leads to asymmetric distribution and the concentration of stress during atomic-scale grinding.
(4) Stress concentration in the material during curve grinding was examined. For *σ_hyd_*, *σ_xx_*, *σ_yy_* and *σ_zz_*, the compressive stress regions were mainly concentrated around the tool, whereas the tensile stress regions were concentrated at the rear of the tool. The peak region for *σ_von_* was not distributed around the tool, but rather, on both sides of the front region of the tool. For *σ_xy_*, the left and right sides of the tool were peak regions of positive shear stress and negative shear stress, respectively.

## Figures and Tables

**Table 1:** MD simulation parameters.

Model	Workpiece	Tool
material	silicon	diamond
dimension	240 Å × 220 Å × 120 Å	side length: 20 Å
lattice	5.432 Å	3.567 Å
number of atoms	315,414	10,930
initial temperature	293 K	
time step	1 fs	
grinding depth	25 Å, 20 Å, 15 Å and 10 Å	
arc radius	0 Å, 20 Å, 40 Å, 60 Å	
grinding velocity	100 m/s	
potential function	Tersoff	

**Table 2:** Simulation parameters of the grinding path.

*R* (Å)	*d_1_* (Å)	*d*_2_ (Å)	Center Coordinate of arc (Å)
0	140	80	(140, −40)
20	120	60	(120, −20)
40	100	40	(100, 0)
60	80	20	(80, 20)
